# CRISPR-Based Biosensors for Medical Diagnosis: Readout from Detector-Dependence Detection Toward Naked Eye Detection

**DOI:** 10.3390/bios14080367

**Published:** 2024-07-28

**Authors:** Kai Hu, Weihong Yin, Yunhan Bai, Jiarui Zhang, Juxin Yin, Qiangyuan Zhu, Ying Mu

**Affiliations:** 1 State Key Laboratory of Industrial Control Technology, Research Centre for Analytical Instrumentation, Institute of Cyber-Systems and Control, Zhejiang University, Hangzhou 310027, China; 12032063@zju.edu.cn (K.H.); 22007060@zju.edu.cn (W.Y.); 3210105967@zju.edu.cn (Y.B.); 3210102508@zju.edu.cn (J.Z.); 2 Academy of Edge Intelligence, Hangzhou City University, Hangzhou 310015, China; yinjuxin@163.com

**Keywords:** CRISPR/Cas system, biosensor, medical diagnosis

## Abstract

The detection of biomarkers (such as DNA, RNA, and protein) plays a vital role in medical diagnosis. The CRISPR-based biosensors utilize the CRISPR/Cas system for biometric recognition of targets and use biosensor strategy to read out biological signals without the employment of professional operations. Consequently, the CRISPR-based biosensors demonstrate great potential for the detection of biomarkers with high sensitivity and specificity. However, the signal readout still relies on specialized detectors, limiting its application in on-site detection for medical diagnosis. In this review, we summarize the principles and advances of the CRISPR-based biosensors with a focus on medical diagnosis. Then, we review the advantages and progress of CRISPR-based naked eye biosensors, which can realize diagnosis without additional detectors for signal readout. Finally, we discuss the challenges and further prospects for the development of CRISPR-based biosensors.

## 1. Introduction

The primary etiologies of global mortality and morbidity can be categorized into acute infectious diseases (including malaria, AIDS, novel coronavirus pneumonia, etc.) caused by bacteria, viruses, fungi, and parasites and non-infectious diseases (including cardiovascular diseases, cancer, diabetes, etc.) caused by the combined effects of genetic, physiological, environmental and behavioral factors [1,2]. When the disease invades the human body, the physiological signals representing the physical condition will change with the progression of the disease. Biomarkers serve as biochemical indicators that can monitor normal biological processes, pathological processes, and pharmacological response processes to therapeutic interventions, as well as any measurable diagnostic indicators [3,4]. Numerous biomolecules can function as biomarkers, including antigens, proteins, nucleic acids, and cells, with nucleic acids and proteins being the most commonly utilized biomarkers [5].

Currently, the primary techniques utilized for biomarker detection include polymerase chain reaction (PCR), recombinase polymerase amplification (RPA), loop-mediated isothermal amplification (LAMP), enzyme-linked immunosorbent assay (ELISA), gel electrophoresis, Western blotting (WB), fluorescence activated cell sorting (FACS), and mass spectrometry analysis [6,7,8,9,10,11,12]. Typically, these methods are employed by skilled professionals with specialized apparatus in isolated laboratory spaces [13,14]. The emergence of biosensors has empowered patients to evaluate their medical status autonomously without the need for a doctor and apparatus. They provide a simple detection method for individuals with limited medical resources [15].

Presently, the exploration of nucleic acids and proteins detection via biosensors has been widely investigated [16,17]. Biosensors typically consist of a biometric element, a transmitter, an amplifier, and a processor. Biometric elements specifically identify targets, transmitters convert biometric signals into alternative measurable signals, and amplifiers and processors are utilized for signal amplification and output [18]. Consequently, the key to improving the detection sensitivity and specificity of biosensors is enhancing the capacity of biological recognition. Moreover, while the highly integrated biosensors have realized the recognition of the target as well as the conversion of the measurable signal, the readout of the output signal still depends on specialized detectors, significantly limiting their application in on-site detection [19,20]. There are nearly 10 million colors that can be distinguished by the human eye, making it a powerful natural detector. A colorimetric biosensor with output that can be observed by the naked eye became a crucial on-site detection tool, thus reducing the dependence of the detector [21,22]. However, the sensitivity of the human eye cannot be compared to that of professional detectors, limiting its ability in the detection of the low-abundance analyte.

The clustered, regularly interspaced short palindromic repeats/CRISPR-associated proteins system (CRISPR/Cas) technology has brought revolutionary advancements in gene editing, which also demonstrates great potential in molecular diagnosis and point-of-care testing (POCT) [23]. Traditional biological recognition predominantly relies on molecular interactions for perception, such as the immune binding of antigens and antibodies and the complementary base pairing of nucleic acid probes. Conversely, the CRISPR/Cas system employs a dual-component recognition mechanism, which is achieved by the combination of a Cas effector and guide RNA, resulting in a superior biological recognition capability [24]. This advantage allows the CRISPR/Cas system to detect a wide range of targets with programmable designs, from proteins to nucleic acids. Consequently, the CRISPR/Cas system has been integrated into biosensors to generate a more sensitive and specific biosensor system [25]. Particularly for the naked eye biosensor, the integration of the CRISPR/Cas system compensates for the limitation of human eye detection sensitivity, significantly lowering the detection limit.

Today, the integration of the CRISPR/Cas system with biosensors provides a rapid, facile, high sensitivity, and specificity-oriented medical diagnosis method. CRISPR-based biosensors relying on the naked eye for signal readout improve the ability of on-site diagnoses without the dependence on professional detectors. In this review, we briefly introduce the principle of CRISPR-based biosensing technology. Subsequently, we review the recent advancements of CRISPR-based biosensors in the field of disease diagnosis, which utilize nucleic acids and proteins as biomarkers. Next, the principle and recent progress of CRISPR-based naked eye biosensors for medical diagnosis are reviewed. Lastly, we discuss the prospects for CRISPR-based biosensors.

## 2. The Principle of CRISPR-Based Biosensors

### 2.1. The Principle of CRISPR/Cas System

CRISPR is originated from the immune defense systems of bacteria and archaea. The CRISPR/Cas system is primarily composed of Cas proteins and CRISPR-derived RNAs (crRNAs) [26]. This system can be categorized into two classes and further subdivided into six types based on the function of the Cas proteins. In the Class I CRISPR/Cas system, multi-subunit effector complexes degrade the target, whereas the Class II CRISPR/Cas system utilizes a single effector to recognize the target [27,28]. The Class II CRISPR/Cas system can be divided into type II (Cas 9), type V (Cas 12), and type VI (Cas 13). Cas 9 and Cas 12 are directly targeted to DNA, while Cas 13 is aimed at RNA targets [29]. Among these CRISPR/Cas system families, Cas 9, Cas 12, and Cas 13 have attracted significant attention in research and are extensively utilized in nucleic acid testing due to the easier assembly of the CRISPR effect complex.

**Figure 1:**
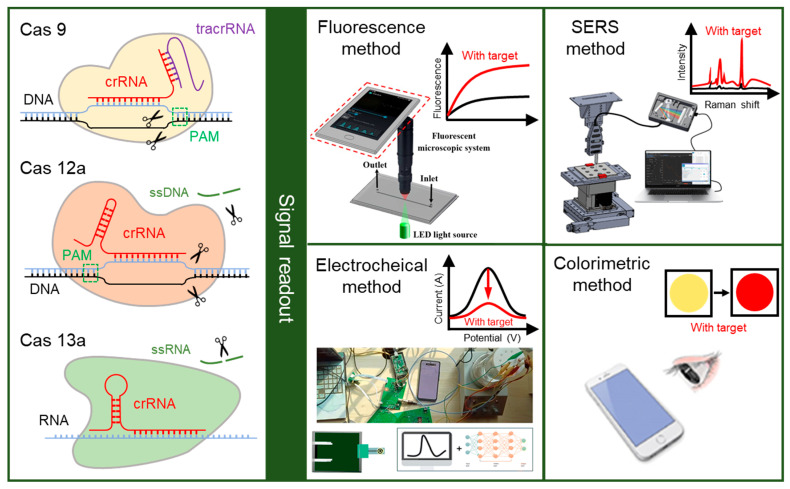
The principle of CRISPR-based biosensors. In the CRISPR-based biosensor, the crRNA in the CRISPR/Cas system (Cas 9, Cas 12a, and Cas 13a) can specifically biological recognize the target, while the Cas protein is used for the cleavage of target and reporter after recognition. The biological signals generated by the cleavage could be converted into other measurable signals, and then read out by fluorescence method, electrochemical method, SERS method, and colorimetric method. Reproduced with permission from Ref. [37], Ref. [38], and Ref. [39]. Copyright 2019 Elsevier. Copyright 2023 Royal Society of Chemistry. Copyright 2024 American Chemical Society.

As illustrated in the left of Figure 1, Cas 9 was the first CRISPR/Cas system employed for gene editing, which consists of *Streptococcus pyogenes* Cas 9 (spCas 9), crRNA and *trans*-activating crRNA (tracrRNA) [30]. When the target double-stranded DNA (dsDNA) is specifically recognized by the crRNA and tracrRNA through a user-defined 20-nt guide RNA sequence, the spCas 9 is activated, resulting in the double-strand break (DSB) in target DNA. Subsequently, spCas 9 cleaves the protospacer adjacent motif (PAM) sequence in the target DNA. The PAM sequence, located at the 3′ downstream of the target DNA, is critical for the binding to Cas 9, which will limit the choice of target DNA sequences due to its high conservatism [31]. Cas 12 is a dual-nuclease with endoribonuclease and endonuclease activities, which was discovered later [32]. Cas 12a, the first discovered Cas 12 enzyme, is used to recognize and cleave the single-stranded DNA (ssDNA) and dsDNA. Different from Cas 9, Cas 12a exhibits target-specific cleavage (*cis*-cleavage) as well as nonspecific nuclear acid cleavage (*trans*-cleavage) [33]. In the CRISPR/Cas 12a system, the crRNA plays a dual role in binding to Cas 12a and recognizing the short T nucleotide-rich PAM sequence in target DNA. Cas 12a is activated and cleaved to both strands of the target DNA after specifically binding to the target nucleic acid fragments under the guidance of crRNA, generating a PAM-distal DNA break with staggered 5′ and 3′ ends. Concurrently, due to the *trans*-cleavage activity, the nonspecific ssDNA is also indiscriminately cleaved [34]. Similar to Cas 12, Cas 13 also exhibits the *trans*-cleavage activity for nonspecific nucleic acids after binding to target RNA. In the Cas 13a system, both the target RNA and non-target RNA are cleaved following the formation of a guide–target RNA duplex, which is realized by a 3′ protospacer flanking site instead of a PAM sequence [35,36]. By modifying the fluorescence group and quenching group at the ends of ssDNA, the biological signals generated by the cleavage of ssDNA can be converted into fluorescence signals. Consequently, the crRNA in the CRISPR/Cas system serves as the biometric element in biosensors for specific recognition of nucleic acid, while the Cas protein serves as the transmitter in biosensors to generate detectable output signals.

### 2.2. The Readout Strategies of CRISPR-Based Biosensors

The CRISPR/Cas system has been used for the diagnosis of various conditions, such as Corona Virus Disease 2019 (COVID-19), pathogens, and cancer, relying on its high sensitivity and specificity [40]. The CRISPR-based biosensors have been developed and applied for medical diagnosis, including antimicrobial resistance in bacterial infections, rapid detection of emerging infectious diseases, and medical early diagnosis [41,42,43,44]. The CRISPR/Cas system is used to recognize the target and cleave the reporter to generate signals. Generally, the detection strategy of CRISPR-based biosensors for nucleic acid detection is to directly recognize target nucleic acid fragments. For the detection of proteins and other small molecules, the specific recognition is indirectly realized by the conversion from non-nucleic-acid targets into DNA intermediates [45]. Then, the signals generated by the cleavage of the reporter are converted into other signals for readouts, such as the fluorescence method, electrochemical method, surface-enhanced Raman spectroscopy (SERS) method, and colorimetric method, which are shown in the right of Figure 1 [46].

The fluorescence readout method in CRISPR-based biosensors uses a fluorescence probe, which is a kind of sequence labeled with a fluorescent group and a quencher group at both ends. In the presence of the target, the Cas protein is activated, and the probes are cleaved by the *trans*-cleavage activity, leading to the separation of the fluorescent group and the quencher group. The concentration of the target can be determined by measuring the intensity of the fluorescence signals generated by the free fluorescent group [47,48]. The detection of fluorescence signals typically relies on commercial apparatuses, such as fluorescence spectrometers and fluorescence microscopes, which are difficult to carry around [49]. A portable smartphone-based fluorescent microscopic system has been designed to read the fluorescence signal from the *Salmonella typhimurium* biosensor, reducing the dependence on heavy apparatus [37].

In the electrochemical readout method, an electrochemical probe is utilized, which can impact the motion of electrons. In the presence of the target, the electrochemical probes on the surface of the electrode are cleaved by the activated Cas protein, inducing an electrochemical signal change through the variation in the transfer distance or number of electrons [50,51]. This electrochemical signal change can be used for quantitative analysis of the target, which is detected by the commercial electrochemical workstation [51]. Integrating the circuit board with a smartphone makes it possible for the miniaturization and portability of the electrochemical workstation, enhancing the on-site detection capability of electrochemical biosensors [38,52].

Surface-enhanced Raman spectroscopy (SERS) is an analytical method utilized for the detection of trace molecules, which can specifically identify unique molecular vibrations of a molecule [53,54]. In the presence of target, the probes on the SERS active surface are cleaved by the activated Cas protein, resulting in a change of SERS signal. This SERS signal change could be detected by the Raman spectrometer and then analyzed by the computer, which is complex and heavy [55]. A portable Raman spectrometer, consisting of two fibers for excitation probe and collection probe and a mini-Raman device, has been proposed [56]. Compared to traditional methods, SERS readout method offers a higher sensitivity and specificity, making it extremely valuable for the detection of gene mutations and low concentrations of analytes [57].

The colorimetric readout method relies on the color change of the biosensor, which is observable to the smartphone or naked eye [39]. When compared to the fluorescence, SERS, and electrochemical signals, this color change is easier to observe without limiting the complex apparatus, making it more suitable for on-site detection [51,58]. The enzymes and nanoparticles that are modified by nucleic acid probes are commonly used in the colorimetric readout method. The color of nanoparticles changes simultaneously with the aggregation and dispersion, and the enzymes catalyze the substrate when they are transferred to the substrate. In the presence of the target, the nucleic acid probes will be cleaved by the Cas protein, and the nanoparticles or enzymes will be released, resulting in an observable color change.

## 3. CRISPR-Based Biosensors for Medical Diagnosis

### 3.1. CRISPR-Based Biosensors for DNA-Targeted Diagnosis

Nucleic acid assumes a pivotal role in organisms by its capacity to archive, encode, and transmit genetic information, which is also closely related to various diseases [59]. Circulating tumor DNA (ctDNA) carries the key genetic information of solid tumors, reflecting the prevalence and recurrence indices of cancer [60,61]. The anomalous expression of miRNA can incite malignant tumors [62,63]. Within genomes associated with COVID-19-related viruses, the envelope protein gene and the nucleocapsid protein gene have been widely used for diagnosis [64]. The CRISPR/Cas system exhibits an impressive capability for the specific biometric recognition of DNA and RNA targets, demonstrating the extensive application of CRISPR-based biosensors in DNA and RNA-targeted diagnosis.

CRISPR/Cas 9, one of the earliest identified CRISPR/Cas systems, has been integrated into biosensors for the detection of dsDNA when complexing with a guide RNA [65]. However, while variants of Cas 9 can target ssDNA, their activity toward dsDNA is significantly weak, ultimately limiting the universality of Cas 9 in DNA detection [66]. The discovery of Cas 12 provides an opportunity for detecting both dsDNA and ssDNA. Remarkably, the activated Cas 12 by target DNA can cleave the bystander ssDNA due to its *trans*-cleavage activity. This cleavage of bystander ssDNA offers an intrinsic signal amplification mechanism, leading to a higher signal intensity than that generated from the Cas 9 system [67,68]. Consequently, many Cas 12-based biosensors have been proposed in medical diagnosis in the past two years.

**Figure 2:**
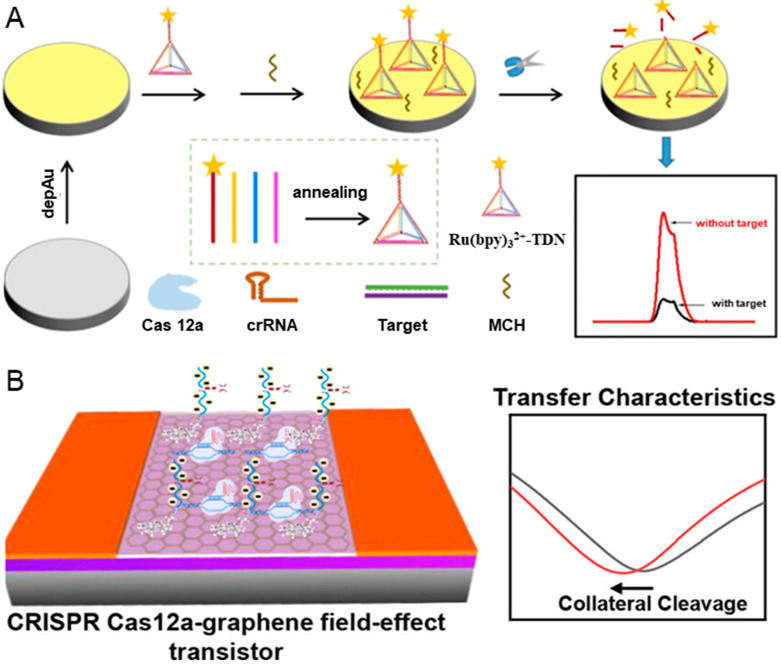
CRISPR-based biosensors for the diagnosis of HPV-16. (**A**) The workflow of CRISPR/Cas 12a-mediated ECL biosensor, including the modification of DNA TDN probes, the cleavage of DNA TDN probes, and signal readout. Reproduced with permission from Ref. [71]. Copyright 2023 American Chemical Society. (**B**) The illustration of CRISPR Cas 12a-gFET biosensor array for ultrasensitive and reliable detection of unamplified DNA. Reproduced with permission from Ref. [72]. Copyright 2023 American Chemical Society.

Cervical cancer ranks among the most common cancers afflicting females [69]. 62% of cervical cancer cases are caused by human papillomavirus 16 (HPV-16), making the detection of HPV-16 crucial for prevention and treatment [70]. In response to the demand for a reliable, simple, accurate, sensitive, and economical HPV-16 detection method, Yu et al. proposed an amplification-free electrochemiluminescence (ECL) biosensor based on CRISPR/Cas system and DNA tetrahedron nanostructures [71]. As shown in Figure 2A, a DNA tetrahedral nanostructure (TDN) is assembled through the annealing of a Ru(bpy)_3_^2+^-labeled ssDNA probe together with three other sequences. Subsequently, TDN is assembled on the electrode surface via the Au-S bond, leaving the signal “turn on”. In the presence of target DNA, the *trans*-cleavage activity of Cas 12 is activated and begins to cleave the TDN, causing the signal “turn off”. The quantitative detection of HPV-16 could be realized according to the change of signal, with the detection limit as low as 8.86 fM.

Nanomaterial has been integrated into CRISPR-based biosensors to improve sensitivity, including quantum dots [73], upconversion nanoparticles [74], magnetic beads [75], and reduced graphene oxide [76]. The graphene field-effect transistor (gFET) has a high sensitivity in identifying biorecognition events occurring at the surface due to its high carrier mobility. Weng et al. reported a CRISPR-Cas 12a-mediated gFET array, which can realize amplification-free, ultrasensitive, and reliable detection of DNA [72]. The *trans*-cleavage activity of CRISPR-Cas 12a is employed to cleave the DNA probes and amplify the biological signals, while the ultra-sensitive gFET is used for signal transmission. As depicted in Figure 2B, the negatively charged ssDNA probes in the graphene surface are cleaved when target DNA exists, resulting in a shift left in the transfer characteristics and charge neutrality point voltage due to the increase in the electron carrier density. Interestingly, the shift left value of charge neutrality point voltage is linearly related to the concentrations of targets. The CRISPR-Cas 12a-mediated gFET array can achieve a detection limit of 1 aM for the ssDNA of HPV-16 synthetic target without the needs of target preamplification, significantly improving detection sensitivity compared to the CRISPR-Cas 9-based gFET biosensor [77].

**Figure 3:**
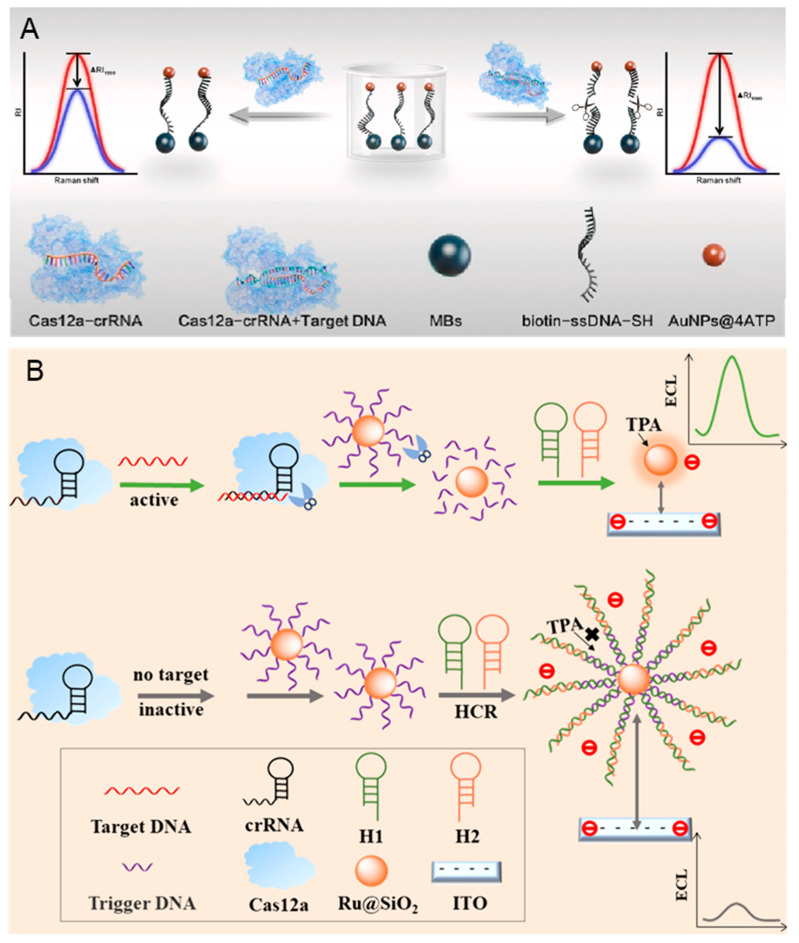
CRISPR-based biosensors for the diagnosis of HBV. (**A**) The principle of amplification-free detection of HBV DNA mediated by CRISPR-Cas 12a using SERS. Reproduced with permission from Ref. [80]. Copyright 2023 Elsevier. (**B**) The principle of the low-background signal-on homogeneous ECL biosensor for HBV detection based on CRISPR/Cas 12a and HCR. Reproduced with permission from Ref. [81]. Copyright 2023 American Chemical Society.

Hepatitis B, caused by hepatitis B virus (HBV) infection, has been a global public health issue. There are more than 250 million individuals who are chronic carriers of HBV DNA, with approximately 0.9 million deaths annually attributable to HBV-related diseases [78]. The monitor of HBV can effectively reduce the risks of cirrhosis and liver cancer [79]. Du et al. developed an amplification-free diagnostic method that uses a CRISPR/Cas 12a-based approach to SERS viral DNA biosensor, which is shown in Figure 3A [80]. The Raman probes (AuNPs@4-ATP) are created by combining gold nanoparticles (AuNPs) modified with the Raman reporter molecule 4-ATP and magnetic spheres, which can maximize the SERS effect and generate differential Raman spectroscopic signals. When the Cas 12 is activated by the target, the ligated AuNPs@4-ATP are cleaved, leading to a decrease in SERS intensity following magnetic separation. This biosensor demonstrates a strong linear relationship between SERS intensity and the DNA concentrations in the range of 0.1 pM–1 nM, with a detection limit of 0.67 pM. However, this method requires magnetic separation before SERS detection, increasing the complexity of the operation.

Reducing the background signal can improve the sensitivity of HBV detection. As shown in Figure 3B, the hybridized chain reaction (HCR) is combined with CRISPR/Cas 12a to develop an ECL biosensor by Luo et al. [81]. The designed DNA probes are modified on the surface of nanoparticles (Ru@SiO_2_NPs), which can be amplified when HCR. In the presence of the HBV DNA, the DNA probes on the Ru@SiO_2_NPs are cleaved due to the *trans*-cleavage activity of Cas 12a, preventing the HCR amplification and generating a strong ECL signal. On the contrary, the ECL signal is low when HBV DNA is absentce. The intensity of the ECL signal demonstrates a strong linear relationship with the HBV concentration range from 10 fM– to 10 nM. Additionally, the detection limit can reach 7.41 fM due to the dual signal amplification.

Beyond diseases caused by viruses, CRISPR-based biosensors have also been utilized for the medical diagnosis of bacterial infections. *Methicillin-resistant Staphylococcus aureus* (MRSA) is a kind of drug-resistant bacteria with significant mortality and morbidity. Wei et al. presented a novel biosensor that can achieve nucleic acid amplification-free ultrasensitive MRSA detection, which is based on the combination of CRISPR/Cas 12a system, on-particle rolling circle amplification, and enzyme-triggered click chemistry [82]. *Mycobacterium tuberculosis* (MTB) is a kind of bacteria that can cause tuberculosis. Chen et al. constructed a magnetic separation-enhanced colorimetry method based on the multiple isothermal amplification and the CRISPR/Cas 14 a system [83]. *Helicobacter pylori* can cause gastric mucosa-associated lymphoid tissue, chronic gastritis, duodenal peptic ulcer disease, and gastric adenocarcinoma. Yu et al. proposed a novel CRISPR/Cas 12a biosensor for the detection of *Helicobacter pylori* based on the amplification of the target by hairpin-mediated self-primer exponential amplification [84].

### 3.2. CRISPR-Based Biosensors for RNA-Targeted Diagnosis

Unlike Cas 9 and Cas 12, which are DNA-targeted, Cas 13 is an RNA-targeted effector exhibiting both *cis*-cleavage and *trans*-cleavage activities [85]. The *trans*-cleavage of Cas 13 has been utilized to cleave FQ-labeled reporter, and the first Cas 13-based nucleic acid detection platform is called Specific High-sensitivity Enzymatic Reporter unLOCKing (SHERLOCK) [86]. SHERLOCK combines RPA or RT-RPA with the CRISPR/Cas 13 system for target amplification and detection, which could be used in the detection of specific strains of Zika and Dengue viruses and the distinction of pathogenic bacteria. Before long, SHERLOCKv2 has been proposed to achieve instrument-free detection, integrating SHERLOCK with four multiplexing channels [87]. Additionally, numerous CRISPR-based biosensors have been proposed for RNA diagnosis in recent years.

**Figure 4:**
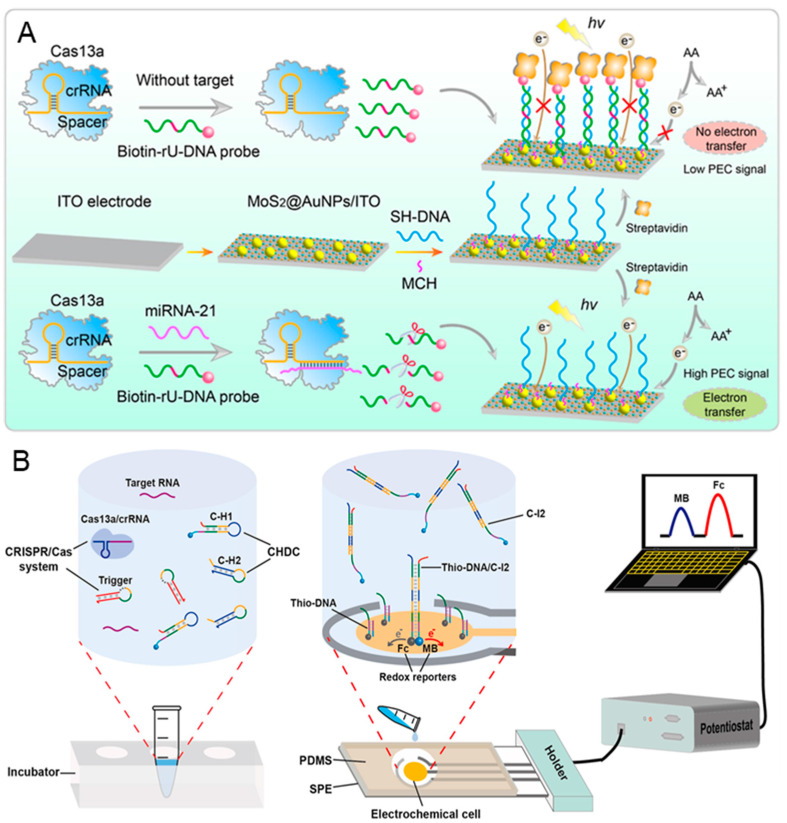
CRISPR-based biosensors for the diagnosis of cancer biomarker miRNA/mRNA: (**A**) The schematic illustration of the proposed Cas-PEC biosensor for the specific and direct assay of miRNA-21. Reproduced with permission from Ref. [91]. Copyright 2023 American Chemical Society. (**B**) The working principle of the Cas-CHDC-powered electrochemical RNA-sensing technology chip. Reproduced with permission from Ref. [92]. Copyright 2021 Elsevier.

MicroRNA (miRNA) and messenger RNA (mRNA) have received widespread attention due to their aberrant expression being closely associated with cancer and cardiovascular disorders [88,89]. However, during the early-stage diagnosis, the abundance of specific miRNA or mRNA can vary from a single molecule to more than 50,000 copies, with the characteristic of easy degradation [90]. CRISPR-based biosensors provide a rapid, highly specific, and sensitive quantification method for miRNA/mRNA. Jiang et al. proposed a novel CRISPR/Cas 13a-mediated photoelectrochemical biosensor for the sensitive and direct analysis of miRNA-21 [91]. As shown in Figure 4A, the thiol-modified capture DNA (SH-DNA) is modified on the surface of MoS_2_@AuNPs that are coated on the ITO electrode. Then, the 6-mercapto-1-hexanol (MCH) is dropped on the electrode to reduce the nonspecific adsorption. In the presence of miRNA-21, the biotin-rU-DNA probes are cleaved due to the *trans*-cleavage activity of Cas 13a protein. Consequently, there are no streptavidin assembles on the electrode because the cleaved biotin-rU-DNA probe cannot hybridize with SH-DNA. This phenomenon enhances the photocurrent signal by improving electron transfer and inhibiting the recombination of photogenerated electrons and holes of MoS_2_@AuNPs. Conversely, the photocurrent signal remains low in the absence of miRNA-21. The proposed CRISPR-biosensor demonstrates a good linear analysis within a range of 1 fM–5 nM, and a lower detection limit of 1 fM.

Due to the insufficient accuracy of single miRNA for early cancer diagnosis, it is necessary to develop a method for detecting multiple miRNA/Mrna [93]. Sheng et al. presented an electrochemical biosensor for high sensitivity sequential measurements of multiple RNAs by integrating the CRISPR/Cas system with a catalytic hairpin DNA circuit (CHDC) [92]. CHDC is an enzyme-free DNA circuit that promotes signal amplification by catalyzed hairpin assembly. The stepwise operation is shown in Figure 4B; the trigger connects the Cas-mediated target detection with the CHDC mechanism. In the presence of target RNA, the triggers are cleaved by the activated Cas 13a, releasing numerous intermediary strands (as the primary amplification). Subsequently, the intermediary strand, C-H1 and C-H2 undergo catalytic cycling through domain hybridization, forming numerous C-H1-C-H2 (C-I2) complexes (as the secondary amplification). Next, C-I2 complexes bind to the thio-DNA when added to chip, thereby enhancing the electrochemical current. This Cas-CHDC-powered electrochemical RNA-sensing technology chip successfully detects six non-small-cell lung carcinoma-related RNAs (miRNA-17, miRNA-155, TTF-1 mRNA, miRNA-19b, miRNA-210, and EGFR mRNA), with results consistent with those obtained from qRT-PCR.

**Figure 5:**
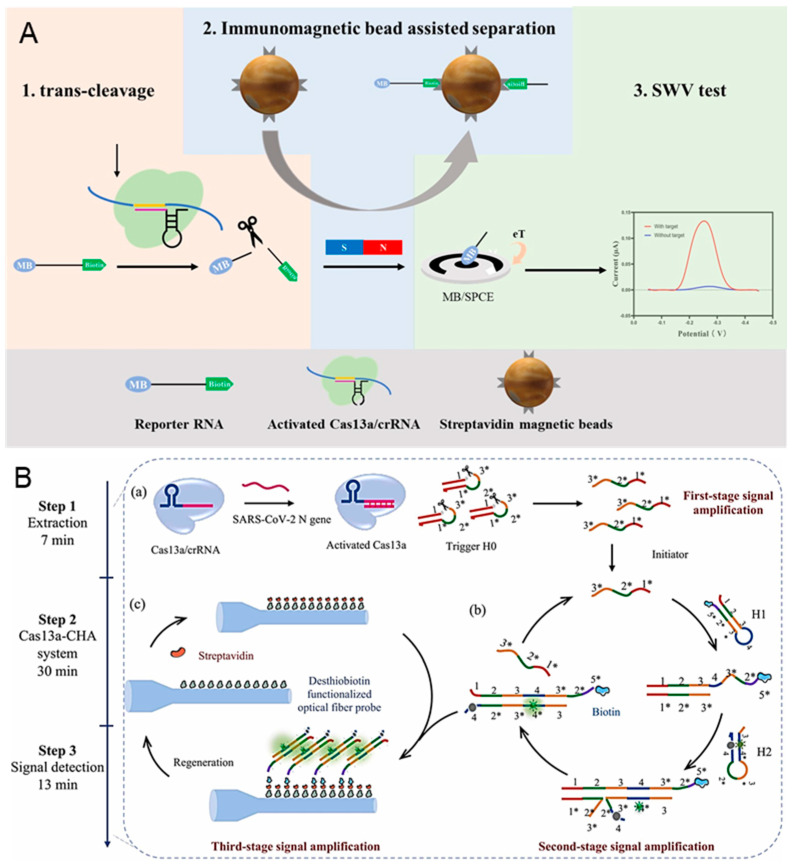
CRISPR-based biosensors for the diagnosis of SARS-CoV-2: (**A**) Principle of the electrochemical CRISPR biosensor for detecting severe acute respiratory syndrome coronavirus 2 (SARS-CoV-2) RNA. (**B**) Schematic illustration of the CRISPR/Cas 13a-powered catalytic hairpin assembly evanescent wave fluorescence biosensor for SARS-CoV-2 detection. (**a**) The cleavage of CHA initiator by the activated Cas 13a. (**b**) The generation of the biotinylated H1/H2 complexes by CHA. (**c**) The capture of biotinylated H1/H2 complexes by the streptavidin-desthiobiotin-functionalized optic fiber probe. Reproduced with permission from Ref. [97]. Copyright 2024 Elsevier.

COVID-19, caused by Severe Acute Respiratory Syndrome Coronavirus 2 (SARS-CoV-2), has become the most serious epidemic due to its strong interpersonal transmission, extensive infection range, and challenging prevention and control [94,95]. The continuous emergence of more infectious SARS-CoV-2 variants has increased the difficulty of prevention due to higher transmission rates and stronger immune escape responses. Han et al. proposed an immunocapture magnetic bead-enhanced electrochemical biosensor for ultrasensitive SARS-CoV-2 detection based on the CRISPR/Cas system [96]. As shown in Figure 5A, a single-stranded RNA (ssRNA) modified with biotin and methylene blue (MB) at both ends is used as the reporter RNA (MB-ssRNA-Biotin). In the presence of target RNA, the target is amplified by the reverse transcription-recombinase-aided amplification (RT-RAA) reaction and the reporter RNA is subsequently cleaved by the activated Cas protein. Next, the streptavidin-coated magnetic beads bind to the cleaved reporter RNA through biotin-streptavidin interaction. The MB is left in the solution after the magnetic separation. Finally, an MB electrochemical signal is detected by the carbon electrode. In the absence of target RNA, the CRISPR/Cas system remains dormant, and the MB electrochemical signal will not be enhanced. The use of magnetic separation can reduce the background noise signal and realize the ultrasensitive detection of SARS-CoV-2 (down to 1.66 aM).

Using the isothermal nucleic acid amplification technologies (like RPA, LAMP, and RAA) for preamplification of SARS-CoV-2 before the detection with a CRISPR-based biosensor can increase the sensitivity but also raise the possibility of nonspecific amplification and false-positive results [98,99]. Song et al. proved a CRISPR/Cas 13a-powered catalytic hairpin assembly (CHA) evanescent wave fluorescence biosensor for target amplification-free SARS-CoV-2 detection [97]. There are three-stage signal amplifications employed in this biosensor, which are shown in Figure 5B. When target RNA exists, the DNA triggers are cleaved by the activated Cas 13a, releasing the intermediate domain of the CHA initiator. Frequently, the downstream CHA system is initiated and generates numerous H1/H2 complexes through multiple catalytic cycles. The biotinylated H1/H2 complexes are then captured by the streptavidin-desthiobiotin-functionalized optic fiber probe. Finally, the target RNA is quantified by detecting the fluorescence signal, which is proportional to the number of H1/H2 complexes. The quantitative SARS-CoV-2 detection is achieved with a detection limit of 18.6 copies/μL, and all detection is finished within 50 min.

### 3.3. CRISPR-Based Biosensors for the Diagnosis of Protein

**Figure 6:**
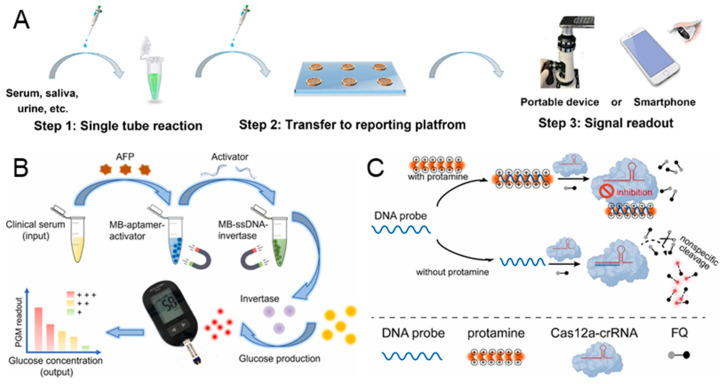
CRISPR-based biosensors for the diagnosis of protein. (**A**) Illustration of workflow for the detection of SARS-CoV-2 nucleocapsid protein by the aptamer-CRISPR/Cas 12a-regulated liquid crystal sensor. The entire workflow can be completed with the following three steps: single-tube reaction, transfer to an LC-based reporting platform, and signal readout. Reproduced with permission from Ref. [39]. Copyright 2024 American Chemical Society. (**B**) The workflow of the CRISPR-powered personal glucose meter biosensing platform for quantitative detection of AFP biomarker. Reproduced with permission from Ref. [104]. Copyright 2023 Elsevier. (**C**) The principle of CRISPR/Cas 12a-based fluorometric biosensor for the detection of protamine, which relies on the electrostatic interaction in DNA probe and protamine. Reproduced with permission from Ref. [105]. Copyright 2023 Elsevier.

Besides nucleic acid sensing, the CRISPR-based biosensor has been applied to the detection of proteins. However, due to the CRISPR/Cas system being nucleic acid-targeted, it is necessary to design a nucleic acid probe to interact with the target protein as a marker before detection [100,101]. Aptamers, which are ssDNA or RNA molecules that can be hybridized with the target protein, have been widely used in the detection of proteins based on biosensors [102,103]. Qi et al. developed an aptamer-CRISPR/Cas 12a-regulated liquid crystal sensor (ALICS) for the ultrasensitive detection of proteins [39]. A DNA probe is designed and modified in the surface of graphene oxide (GO), which contains an aptamer sequence to capture the target protein and an activation sequence to activate the CRISPR/Cas 12a system. As shown in Figure 6A, in the presence of a target protein, the DNA probes are released from the GO when their aptamer sequence successfully binds to the target protein. Then, the Cas 12a is activated by the activation sequence from the released DNA probes and introduced to the LC-based reporting chip. Consequently, the ssDNA on the LC is cleaved, resulting in an optical change from bright to dark. The detection limit of ALICS can reach 0.4 pg/mL for SARS-CoV-2 nucleocapsid protein and 20 pg/mL for carcino-embryonic antigen. Moreover, the signal readout is achieved by a portable device or a smartphone, making it particularly suitable for point-of-care applications.

Alpha-fetoprotein (AFP) in serum is considered as an important protein biomarker for early-stage diagnosis of liver cancer [106]. Jia et al. proposed a simple, affordable, and portable CRISPR-powered personal glucose meter biosensing platform for the quantitative detection of the AFP biomarker in serum samples [104]. The activator with the AFP aptamer is modified on the surface of the magnetic bead, forming the MB-aptamer-activator probe. The ssDNA-conjugated invertase is immobilized on the magnetic bead, referred to as MB-ssDNA-invertase. In the presence of AFP, the activator is released due to the binding of AFP and AFP aptamer, which is shown in Figure 6B. After the first magnetic separation, the Cas 12a is activated by the released activator in the supernatant, leading to the cleavage of the MB-ssDNA-invertase and the release of free invertase. Following a second magnetic separation, the sucrose solution is hydrolyzed into glucose with the incubation of invertase. Finally, the glucose is detected by a simple glucose meter, and the AFP is quantified simultaneously. While using this proposed CRISPR-based biosensor, we noted that the AFP sample can be detected at a concentration as low as 10 ng/mL.

Although the aptamer is a versatile recognition element that can specifically bind to different proteins, its binding efficacy can be easily affected by the chemical environment [107]. Therefore, some CRISPR-based biosensors have been developed to target proteins without the use of aptamers. Protamine is a medicinal protein that is widely used after surgery to reverse the anticoagulant effects of heparin and can cause hypertension, bradycardia, and dyspnea if used excessively [108]. In order to monitor the concentration of protamine without aptamers, Ji et al. reported a CRISPR/Cas 12a-based fluorometric biosensor for the detection of protamine, relying on the electrostatic interaction [105]. The substrate DNA (DNA probe) binds to the positively charged protamine through strong electrostatic interactions. As shown in Figure 6C, in the absence of protamine, the DNA probe activates Cas 12a, resulting in the cleavage of the DNA reporter and the generation of fluorescence signals. Conversely, when protamine exists, the DNA probe tightly binds to the protamine, reducing the activation of Cas 12a and generating a lower fluorescence signal. With the increase in protamine concentration, the fluorescence signal decreases linearly, ranging from 0.04 μg/mL to 3.2 μg/mL. Furthermore, the detection limit is 0.03 μg/mL and an entire sample-to-response time of about 1 h.

## 4. CRISPR-Based Naked Eye Biosensors for Medical Diagnosis

The emergence of biosensors decreases the reliance on apparatuses for biosensing but requires specialized detectors for signal readout, like fluorescence spectrophotometers, Raman spectrometers, and electrochemical workstations. The colorimetric readout method relies on visual color changes observable by the naked eye, thus offering the advantages of detector independence, visibility, simplicity, and on-site implementation [109]. However, the sensitivity of the human eye is considerably lower than the sophisticated instrumental transducers, notably limiting the ability to quantify the lower analyte concentrations with naked eye biosensors [110,111]. Integrating the CRISPR/Cas system into naked eye biosensors enhances their analytical performance in medical diagnosis [112].

**Figure 7:**
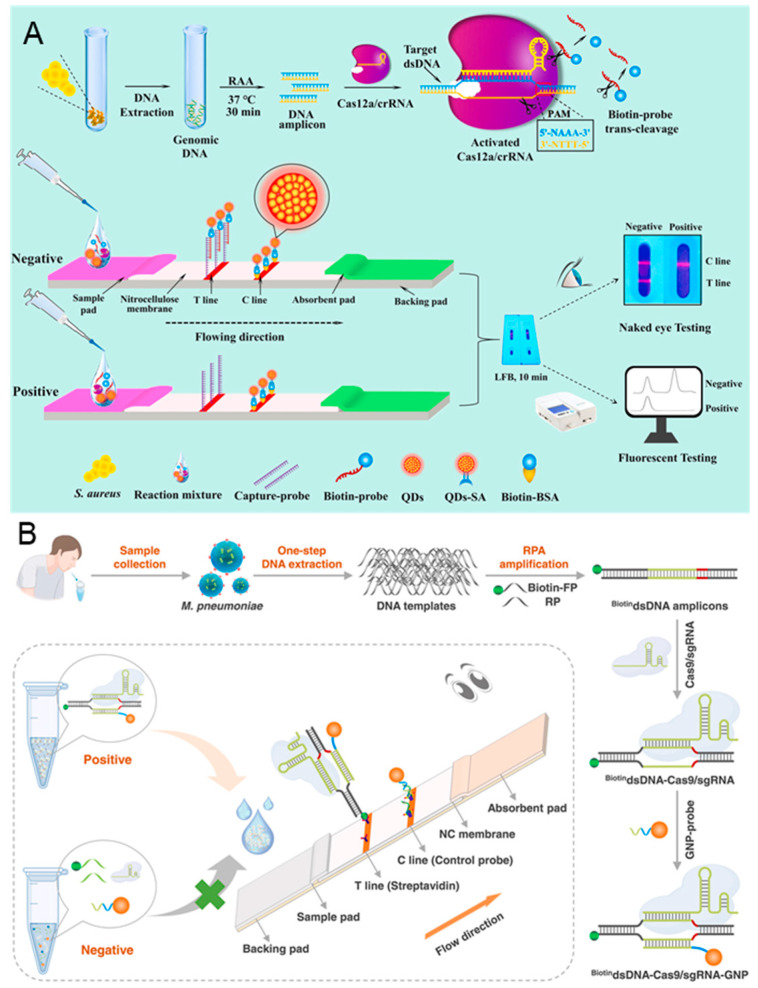
CRISPR-based lateral flow biosensors for medical diagnosis. (**A**) Principle of CRISPR/Cas 12a-based fluorescence-enhanced lateral flow biosensor. The detection workflow can be divided into DNA extraction, RAA amplification, CRISPR/Cas 12a recognition, and LFB detection. Reproduced with permission from Ref. [73]. Copyright 2021 Elsevier. (**B**) Schematic illustration of CRISPR/Cas 9 LFB; the sample can be detected after DNA extraction, RPA amplification, CRISPR/Cas 9 recognition, GNP-probe binding, and LFB detection. Reproduced with permission from Ref. [114]. Copyright 2023 Elsevier.

Lateral flow biosensor (LFB) is the most successful commercialized testing tool, owing to its advantages in low cost, fast response, simple operation, and strong compatibility [113]. The LFB consists of a sample pad, a conjugated pad, an absorbent pad, a test pad, and a backing pad. The sample and biorecognition elements are placed on the sample and conjugated pads, while the detection occurs on the test pad. The absorbent pad generates the driving force for the analytes, while the backing pad provides the support. As shown in Figure 7A, Zhou et al. reported a novel CRISPR/Cas12a-based fluorescence-enhanced LFB in conjunction with functionalized quantum dots (QDs), combined with recombinase-assisted amplification (RAA), to establish low-cost, simple, and sensitive detection of *Staphylococcus aureus* (*S. aureus*) [73]. Streptavidin (SA) modified QDs (QDs-SA) are synthesized to enhance fluorescence signal, biotin probes are designed as the special target capture probes, and capture probes are bind to the T line to capture the biotin probes. In the presence of *S. aureus* DNA, the target DNA is amplificated by RAA and then activated the *trans*-cleavage activity of Cas 12a, resulting in the cleavage of the biotin probe. The cleaved biotin probe cannot be captured by the capture probe, preventing the formation of QDs-SA-biotin-probe-capture-probe. There is no signal accumulation on the T line, while the C line exhibits an obvious fluorescent signal. In the absence of *S. aureus* DNA, the comparable fluorescence signal intensities on the T and C lines can be observed. Without enrichment culture, the detection limit can reach 75 aM and the detection can be finished within 70 min.

Naked eye LFB biosensors are widely used for COVID-19 detection, which are also adapted for the detection of *Mycoplasma pneumoniae* (*M. pneumoniae*). *M. pneumoniae* is a common cause associated with acute respiratory infections in humans. Accurate diagnosis of *M. pneumoniae* is critical to prevent and monitor outbreaks of acute respiratory infections. As shown in Figure 7B, Zhu et al. proposed a point-of-care LFB based on the recognition property of CRISPR/Cas 9 for the detection of *M. pneumoniae* [114]. A biotinylated forward primer (biotin-FP) is designed for the RPA amplification of *M. pneumoniae* DNA, and a gold nanoparticle probe (GNP-probe) is synthesized for signal amplification. In the presence of target DNA, numerous biotin DNA amplicons are generated after RPA, which are recognized by CRISPR/Cas 9. The homologous sequence (~20 nt) in amplicons is then replaced by GNP-probe, forming the biotin-DNA-Cas 9-GNP complex. The complex can bind to the streptavidin modified on T line, leading to a red color due to the accumulation of GNPs. On the contrary, there is no biotin-DNA generated in the absence of target DNA. The free GNP-probes are captured by the pre-immobilized C line. The entire detection, including DNA extraction, RPA amplification, CRISPR/Cas 9 recognition, and visual analysis, can be completed in 30 min, with the detection limit reaching 3 copies/test.

**Figure 8:**
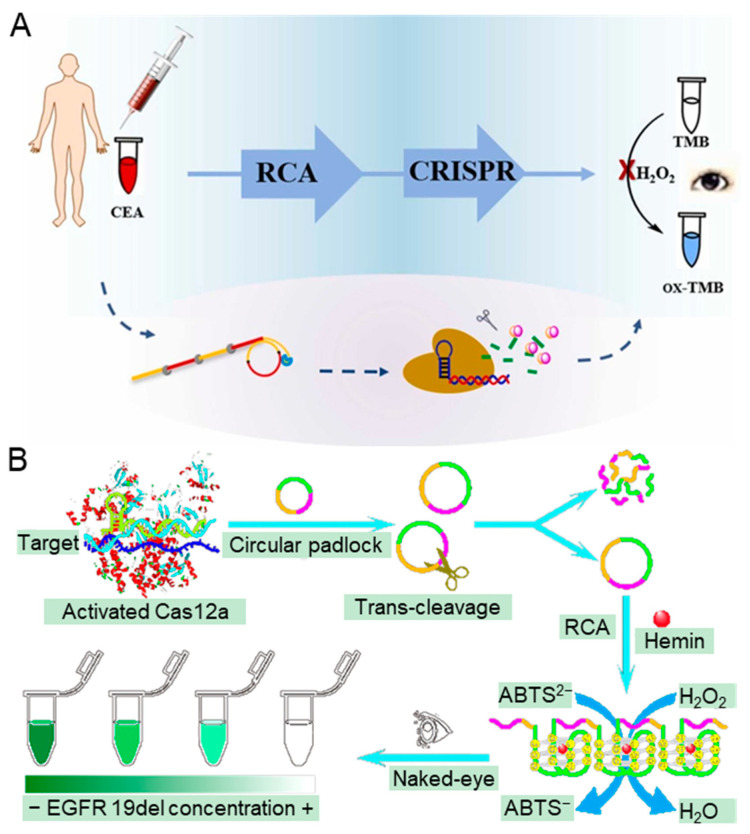
CRISPR-based biosensors for medical diagnosis relying on observable color changes. (**A**) The principle of the colorimetric method for CEA based on the catalytic activity of peroxidase of DNA-Ag/Pt NCs, RCA, and CRISPR/Cas12a. The detection can be divided into CEA binding, RCA amplification, CRISPR/Cas recognition, and TMB signal conversion. Reproduced with permission from Ref. [116]. Copyright 2022 Elsevier. (**B**) The workflow of the naked eye analysis strategy via CRISPR/Cas 12a-triggered RCA for EGFR 19del gene mutation detection. The approach involves CRISPR/Cas recognition, RCA amplification, and ABTS^2−^ signal conversion. Reproduced with permission from Ref. [117]. Copyright 2022 American Chemical Society.

In recent years, nanomaterials with enzyme-like catalytic activity have been widely used in CRISPR-based biosensors that can be observed by the naked eye [115]. As shown in Figure 8A, Mu et al. reported a novel colorimetric biosensor utilizing the catalytic activity of peroxidase-like chromogenic reaction of DNA-Ag/Pt nanoclusters (DNA-Ag/Pt NCs) for the detection of carcinoembryonic antigen (CEA) [116]. The DNA-Ag/Pt NCs are used for the catalysis of H_2_O_2_ to oxidize 3,3,5′,5′-tetramethyl benzidine (TMB), generating the blue substance ox-TMB. In the presence of CEA, target CEA will bind to the aptamer and release the primer chain, which is amplificated by the rolling circle amplification (RCA). The amplicons can activate the *trans*-cleavage activity of Cas 12a and then cleave the DNA-Ag/Pt NCs, preventing the catalysis of TMB. Conversely, the TMB will be catalyzed to produce the blue substance by the complete DNA-Ag/Pt NCs when target CEA absents. This visible biosensor exhibits high sensitivity for CEA, with a linear range from 2.5 pg/mL to 2.0 ng/mL and a detection limit of 0.94 pg/mL. The integration of RCA and CRISPR/Cas 12a has also been employed to detect the epidermal growth factor receptor (EGFR), which is shown in Figure 8B [117]. In the presence of EGFR 19del, the Cas 12a is activated and begins to cleave the designed circular padlocks. The cleaved random fragments cannot initiate RCA, resulting in a colorless solution. When the target is absent, the designed circular padlocks become the templates to initiate RCA to generate long ssDNA, which can be folded into G-quadruplex/hemin DNAzymes, resulting in the catalysis of the oxidation of 2,2′-azino-bis diammonium salt (ABTS^2−^). An obvious color change can be observed by the naked eye. This method demonstrates a robust selectivity and anti-interference ability, with a detection limit as low as 20 fM.

CRISPR-based naked eye biosensors have been widely applied in the diagnosis of nucleic acids and proteins, eliminating the additional readout detectors. However, due to the human eye’s limited sensitivity when compared to professional detectors, the detection limit of the naked eye method is lower than that of the electrochemical method and fluorescence method. Table 1 summarizes the latest biosensors for medical diagnosis, detailing their medical application, detected target, Cas protein type, detection limit, and readout detector. Both RNA and proteins have been employed as targets for the diagnosis of COVID-19 by CRISPR-based biosensors, achieving a minimal detection limit of 1 copy/μL, with results readable by the naked eye. For other viral diagnostics, such as HPV and HIV, the minimal detection limit has also been reduced to a single nucleic acid molecule per microliter of the sample, whose signal readout still relies on specialized detectors. Proteins are commonly utilized as targets in cancer and disease diagnostics, with a minimal detection limit of 1 fg/mL that could be read out by fluorescence spectrophotometer. In the CRISPR-based naked eye biosensors, the minimal detection limit for nucleic acids is 1 copy/μL, whereas for proteins, it can reach 0.94 pg/mL.

## 5. Prospects for CRISPR-Based Biosensors

Timely detection and monitoring of biomarkers (such as DNA, RNA, and protein) are crucial for medical diagnosis. Consequently, biosensors have emerged as a valuable tool for detecting biomarkers, which can ignore the limitation of specialized equipment and skilled operators [25]. The advent of CRISPR/Cas-based biotechnology has proven to be a powerful and accessible tool for enhancing biosensing strategies due to its highly selective sensing mechanism [34,128]. Additionally, the collateral cleavage activity of the Cas protein can amplify the signal and enhance the detection sensitivity [129]. The development of the CRISPR/Cas-based nucleic acid detection method, like SHERLOCK, one-HOur Low-cost Multipurpose highly Efficient System (HOLMES), and DNA endonuclease-targeted CRISPR trans reporter (DETECTR), symbolizing the beginning of this innovative type of biosensing technology in the diagnosis field [67,86,130]. The CRISPR-based biosensor integrated with CRISPR/Cas recognition system provides a rapid, in-field, sensitive, specific quantitative assay capable of detecting specific sequences. The Cas 9, Cas 12, and Cas 13 effectors have been extensively applied in biosensors for the detection of DNA and RNA. The incorporation of aptamer expends the detection targets of CRISPR-based biosensors to proteins, small molecules, exosomes, and bacteria [131]. Furthermore, the CRISPR-based biosensor can meet various detection requirements by employing different signal readout methods such as the fluorescence method, electrochemical method, SERS method, and colorimetric method. The CRISPR-based biosensors based on the colorimetric method are more suitable for on-site detection because the generated color change signals can be detected by the naked eye. Based on these advancements, the application of CRISPR-based biosensors in medical diagnosis is continually expanding.

However, there are still many challenges that remain for the development of CRISPR-based biosensors: (1) The discovery of the Cas protein family is still incomplete, with many new Cas proteins yet to be identified. Harrington et al. discovered Cas 14, which can be used to bind and cleave the target of ssDNA [132]. Cas 14 is more specific than Cas 12a in recognizing ssDNA, showing great potential in biosensing technology [133]. (2) The application of CRISPR-based biosensors for detecting non-nucleic-acid targets is still very limited due to the lack of a universal method. The aptamer is used to bind the non-nucleic-acid target for signal conversion, but this approach is constrained by a narrow range of targets. (3) The sensitivities of the CRISPR-based biosensors need to be improved. Integrating more signal amplification method into CRISPR-based biosensors can improve the detection sensitivity, such as the isothermal amplification technologies (like RPA, LAMP and HCA) [72,91,134] and signal amplification nanomaterials (like AuNPs, MXene, and GO) [80,82,135]. Additionally, reducing background can also improve the sensing performance, which is generated by the long reaction time [81,136]. (4) The storage of CRISPR-based biosensor is challenging because the proteins used in the detection system cannot be stored at room temperature. The use of a freeze-drying strategy can solve this problem, but the activity of thermal enzymes used for amplification might be limited [137].

Overall, CRISPR-based biosensors have demonstrated significant potential as a fast, efficient, and portable monitor for medical diagnosis, with sensitivity comparable to PCR in vitro analysis. The CRISPR-based naked eye biosensors provide a solution to reduce the reliance on detectors, increasing their potential for on-site detection. These CRISPR-based biosensors not only target nucleic acid detection but also begin to expand to other non-nucleic-acid targets. We anticipate that continuing advancement and research in CRISPR-based biosensors would further revolutionize the development of biomarker detection strategies. In the future, CRISPR-based biosensors are expected to play increasingly important roles in medical diagnosis and other medical fields.

## Figures and Tables

**Table 1:** Overview of up-to-date biosensors for medical diagnosis.

Medical Application	Target	Cas Protein	Detection Limit	Readout Detector	Reference
non-small-cell lung carcinoma	miRNA	Cas 13a	30 copy/μL	electrochemical workstation	[92]
cardiovascular diseases	protein	Cas 12a	10 pg/mL	electrochemical workstation	[118]
cancer and inflammation	miRNA	Cas 12a	6 × 10^3^ copy/μL	fluorescence spectrometer	[44]
COVID-19	RNA	Cas 13a	3 copy/μL	naked eye	[119]
COVID-19	protein	Cas 12a	1.5 pg/mL	electrochemical potentiostat	[120]
COVID-19	RNA	Cas 13a	1 copy/μL	electrochemical workstation	[96]
COVID-19	RNA	Cas 13a	18.6 copy/μL	fluorescence platform	[97]
COVID-19	protein	Cas 12a	0.4 pg/mL	smart phone	[39]
COVID-19	RNA	Cas 12a	1 copy/μL	naked eye	[121]
COVID-19	RNA	Cas 12a	22.5 copy/μL	naked eye	[122]
*Mycoplasma pneumoniae*	DNA	Cas 9	1.5 copy/μL	naked eye	[114]
human papilloma virus	DNA	Cas 12a	5.3 × 10^3^ copy/μL	electrochemical workstation	[71]
human papilloma virus	DNA	Cas 12a	0.6 copy/μL	semiconductor device analyzer	[72]
human papilloma virus	DNA	Cas 12a	0.02 ng/μL	fluorescence microscope	[123]
human immune-deficiency virus	RNA	Cas 13a	100 copy/μL	personal glucose meter	[124]
human immune-deficiency virus	RNA	Cas 13a	1.9 copy/μL	fluorescence device	[125]
hepatitis B virus	DNA	Cas 12a	6 × 10^4^ copy/μL	Raman spectrometer	[80]
hepatitis B virus	DNA	Cas 12a	4.4 × 10^3^ copy/μL	electrochemical workstation	[81]
early cancer screening	miRNA	Cas 13a	600 copy/μL	electrochemical workstation	[91]
early cancer screening	miRNA	tandem Cas 13a/Cas 12a	1.8 × 10^5^ copy/μL	naked eye	[112]
early cancer screening	protein	Cas 12a	0.94 pg/mL	naked eye	[116]
early cancer screening	DNA	Cas 12a	1.2 × 10^4^ copy/μL	naked eye	[117]
liver cancer	protein	Cas 12a	10 ng/mL	personal glucose meter	[104]
acute kidney transplant rejection	protein	Cas 12a	18 pg/mL	naked eye	[126]
antidote for heparin	protein	Cas 12a	0.03 μg/mL	multifunctional plate reader	[105]
breast cancer	protein	Cas 12a	1 fg/mL	fluorescence spectrophotometer	[127]
pathogenic bacteria	DNA	Cas 12a	540 CFU/mL	naked eye	[73]

## Data Availability

Not applicable.
